# A Dopaminergic Gene Cluster in the Prefrontal Cortex Predicts Performance Indicative of General Intelligence in Genetically Heterogeneous Mice

**DOI:** 10.1371/journal.pone.0014036

**Published:** 2010-11-17

**Authors:** Stefan Kolata, Kenneth Light, Christopher D. Wass, Danielle Colas-Zelin, Debasri Roy, Louis D. Matzel

**Affiliations:** Department of Psychology, Rutgers University, Piscataway, New Jersey, United States of America; Duke University, United States of America

## Abstract

**Background:**

Genetically heterogeneous mice express a trait that is qualitatively and psychometrically analogous to general intelligence in humans, and as in humans, this trait co-varies with the processing efficacy of working memory (including its dependence on selective attention). Dopamine signaling in the prefrontal cortex (PFC) has been established to play a critical role in animals' performance in both working memory and selective attention tasks. Owing to this role of the PFC in the regulation of working memory, here we compared PFC gene expression profiles of 60 genetically diverse CD-1 mice that exhibited a wide range of general learning abilities (i.e., aggregate performance across five diverse learning tasks).

**Methodology/Principal Findings:**

Animals' general cognitive abilities were first determined based on their aggregate performance across a battery of five diverse learning tasks. With a procedure designed to minimize false positive identifications, analysis of gene expression microarrays (comprised of ≈25,000 genes) identified a small number (<20) of genes that were differentially expressed across animals that exhibited fast and slow aggregate learning abilities. Of these genes, one functional cluster was identified, and this cluster (Darpp-32, Drd1a, and Rgs9) is an established modulator of dopamine signaling. Subsequent quantitative PCR found that expression of these dopaminegic genes plus one vascular gene (Nudt6) were significantly correlated with individual animal's general cognitive performance.

**Conclusions/Significance:**

These results indicate that D1-mediated dopamine signaling in the PFC, possibly through its modulation of working memory, is predictive of general cognitive abilities. Furthermore, these results provide the first direct evidence of specific molecular pathways that might potentially regulate general intelligence.

## Introduction

While the behavioral correlates of general intelligence have been extensively studied, an elucidation of the neural or molecular determinants of this trait has been slow, despite its having been described as the “holy grail” of intelligence research [1]. Recent progress has been made, however, using functional brain imaging studies. For the most part these studies have suggested that the same brain regions that are engaged by working memory tasks, e.g., prefrontal cortex (PFC), are also recruited during performance on intelligence tests [2]–[5]. These results converge with a larger body of research to suggest that working memory (and in particular, its reliance on selective attention) and general intelligence are closely related constructs [6]–[10]. Despite this progress, a more complete understanding of the molecular and cellular networks that are involved in general intelligence has to some degree been hindered by restrictions on work with human subjects (where for instance, it is typically not possible to assess gene expression in brain tissue). To mitigate this impediment, we used recently developed methods to assess the general cognitive abilities (here termed general learning abilities) of laboratory mice.

We have previously reported the existence of a general learning factor in mice that is structurally analogous to general intelligence in humans [11]–[12] Specifically, we observed that when genetically heterogeneous mice were assessed on a battery of learning tasks (e.g., Lashley III maze, passive avoidance, spatial water maze, odor discrimination, fear conditioning) designed to tax different sensory/motor, information processing, and motivational systems, approximately 30–40% of the variance in performance across the tasks could be explained by a single factor. This factor was determined to be independent of stress reactivity and sensory or motor abilities, as variations in these modalities did not load on a general learning factor or correlate with individual animals' performance in the learning battery [13]. Directly modulating stress reactivity through pharmacological means (i.e., chlorodiazepoxide) also did not change the structure of the factor [14]. Furthermore, this cognitive trait was determined to share properties of human intelligence, including a reliance on working memory and selective attention [15]–[16].

In order to elucidate the molecular pathways related to general intelligence, here we characterized the gene expression patterns in the PFC of mice with high general learning abilities relative to those of mice with low general learning abilities in an effort to identify functional clusters of genes that may underlie this cognitive trait. Due to the close relationship between PFC activity and performance on intelligence batteries, we focused this analysis on gene expression in the PFC. We hypothesized that genes related to working memory capacity and selective attention would be differentially expressed in these two groups of animals (owing to the close relationship between working memory and general intelligence). Furthermore, in an attempt to find a direct relationship between gene expression and general intelligence, having identified differentially expressed genes, we then assessed expression levels for those target genes in a population of 50 mice, whose general learning abilities had been previously quantified. This strategy allowed us to directly explore the relationship between specific molecular pathways and general intelligence.

## Methods

### Subjects

CD-1 mice exhibit considerable behavioral variability, and thus are particularly well suited for studies of individual differences. These mice are an outbred strain that was derived in 1926 from an original colony of non-inbred Swiss mice consisting of 2 males and 6 females. Estimates of genetic variation in this line have indicated that after 50 years of breeding, they remained very similar to wild mouse populations [17]. For this study, 60 male CD-1 mice (two replications of 30 mice each) were obtained from Harlan Sprague Dawley (Indianapolis, IN). The mice arrived in our laboratory between 66–80 days of age, and ranged from 25–34 grams at the start of testing. Testing began when the mice were 90–110 days of age, an age which corresponds with young adulthood. The mice were housed individually in clear shoebox cages in a temperature and humidity controlled colony room and were maintained on a 12 h light/dark cycle. In order to minimize any effect of individual differences in stress reactivity to handling, prior to the start of the experiment all of the animals were handled for 90 sec/day, five days/week over a period of two weeks prior to the start of behavioral testing.

### Behavioral Methods

The 60 CD-1 mice used in this replication were assessed (in 2 independent replications) on five learning tasks (i.e., the Lashley III maze, passive avoidance, spatial water maze, associative fear conditioning and odor guided discrimination) which have previously constituted the core tasks used to evaluate general learning abilities. These tasks were chosen so that they place unique sensory, motor, motivational, and information processing demands on the animals. Thus the only commonality between these tasks is that which is most general (i.e., a general learning ability). Briefly, passive avoidance is an operant conditioning task in which animals must learn to suppress a native behavioral tendency (movement off an elevated platform) in order to avoid aversive light and noise stimulation. The spatial water maze encourages animals to integrate spatial information to efficiently escape from a pool of water. Odor discrimination is a task in which animals must discriminate and use a target odor to guide their search for food. Lastly, fear conditioning (assessed by behavioral “freezing”) is a conditioning test in which the animals learn to associate a tone with the presentation of a shock. In all of these tasks the animals were trained well past the point of asymptotic performance. In this way the total amount of learning was equated as much as possible between all the animals. This was done so as to minimize any affect the extent of learning might have on gene expression. Each of these five tasks is described in detail below.

### Lashley III Maze (LM)

This maze consisted of a start box, three interconnected alleys and a goal box. Previous studies have shown that the latency to find the goal box and the number of wrong turns and re-tracings decreased over successive trials. When extra-maze cues are minimized, the animals tend to use egocentric methods to locate the goal box (e.g., fixed motor patterns).

A Lashley III maze, scaled for use with mice, was constructed from black Plexiglas and located in a dimly lit room (10 Lux at the floor of the maze). A 3 cm diameter white circle was located in the center of the goal box, and a 45 mg Bio-serv food pellet (dustless rodent grain) was placed in the cup to motivate the animal's behavior.

Food-deprived animals were acclimated and trained on two successive days. Prior to acclimation they were exposed to three pellets of the reinforcer in their home cage. On the acclimation day, each mouse was confined in each of the first three alleys of the maze for 4 min and the final alley, wherein three food pellets were placed in goal box, for 6 min. At the end of each period, the animal was physically removed from the maze and placed in the next alley. This was done so as to acclimate the animals to the apparatus prior to training/testing. On the training days, the animals were placed in the start box and allowed to freely navigate the maze during which time the number of errors (wrong turns and re-tracings absent a turn) were recorded. Upon consuming the pellet, the animals were returned to their home cage for an 18 min inter-trial interval during which time the maze was cleaned. The animals completed five trials during the first day of training and three trials on the second.

### Passive Avoidance (PA)

In this assay, animals learned to suppress their exploratory tendency in order to avoid aversive stimuli. The animals were placed on a platform and upon stepping down they were exposed to an aversive compound stimulus consisting of a bright light and loud oscillating tone (i.e., “siren”).

A chamber with a white grid floor 16×12 cm (l× w), illuminated by a dim light, was used for both acclimation and testing. An enclosed platform (70×45×45 cm, l× w× h) constructed of black Plexiglas and elevated 5 cm above the grid floor was located at the back of the chamber. There was only one opening from the platform facing the grid floor which allowed the animal to step down onto the floor. The exit from the platform could be blocked remotely by a clear Plexiglas guillotine-style door. When an animal left the platform and made contact with the grid floor, the aversive stimulus compound was initiated. The tone (80 dBc above a 50 dBc background, 2.4–3.7 kHz) was generated by a piezoelectric buzzer (RadioShack, 273-057) and the light was generated by a 100W halogen flood light (located 14 cm from the base of the platform).

During training, the animals were placed on the platform with the door closed, confining them in the enclosure. After 5 min, the door was opened and the latency of the animal to leave the platform and make contact with the floor was recorded. After they made contact, the aversive stimuli were initiated and the door was lowered, exposing them to the stimuli for 4 sec, after which they were allowed access to the enclosure again. This procedure was repeated for two additional trials. For purpose of ranking the animals, the ratio of the step down latency on the second trial to step down latency on the first trial (prior to any learning) served as the index of learning.

### Spatial Water Maze (WM)

This task required the animals to locate a submerged platform in a pool of opaque water. Absent distinct intra-maze cues, animals' performance in this maze is highly dependent on the integration of extra-maze spatial cues. The animals are motivated by their aversion to water. The latency and the path length to locate the platform decrease over successive trials, despite entering the pool from different locations.

A round pool (140 cm diameter, 56 cm deep) was filled to within 20 cm of the top with water that is clouded with a nontoxic, water soluble black paint. A hidden 14 cm diameter black platform was located in a fixed position 1 cm below the surface of the water. The pool was enclosed by a ceiling high black curtain on which five different light patterns (which served as spatial cues) were fixed at various positions.

On the day prior to training, each animal was confined to the platform for 360 sec. by a clear Plexiglas cylinder that fits around the platform. On the next two training days, the animals were started from one of three positions for each trial such that no two subsequent trials start from the same position. The animal is said to have successfully located the platform when it places all four paws on the platform and remains for 5 sec. After locating the platform or swimming for 90 sec, the animals were left or placed on the platform for 10 sec. They were then removed for 10 min. and placed in a holding box before the start of the next trial. Each animal completed 14 total trials (six on the first training day, and four on each of the following two days). The latency to find the platform was recorded for each trial. During the first replication the path length distances to locate the platform were also recorded using custom Matlab software (Mathworks, Natick, MA).

### Associative Fear Conditioning (FC)

In this task the animals received a tone (CS) paired with a mild foot shock (US), after which the animals exhibit fear of the tone as evidenced by “freezing” during its presentation. The training box was contained within a sound- and light-attenuating chamber. This training box (16.5×26.5×20 cm) was brightly lit with a clear Plexiglas front/back, and one stainless steel and one clear Plexiglas side wall. The floor was composed of a steel grid (5 mm spacing) from which a 0.6 mA constant current footshock could be delivered from a shock scrambler (Lafayette Instruments, Lafayette, IN). The tone CS (60 dB, 2.9 kHz) was delivered by a piezoelectric buzzer (Med Associates, EV-203a).

The animals were acclimated to the training context by placing each animal in the box for 20 min on the day prior to training. Training on the subsequent day occurred in a single 18 min session during which the animals received three noise-shock pairings after 6 min, 10 min, and 16 min. The CS presentation consisted of a pulsed (0.7 sec on, 0.3 sec off) 20 sec. tone. Coincident with the offset of the tone, the shock (US) was presented for 500 msec.

To quantify the conditioned fear responses, the animals were videotaped and both the time spent freezing 20 sec prior to the initiation of the tone as well as during the tone were scored by an independent observer. Freezing was defined as no movement of the front or hind feet exceeding 5 mm (the distance between the floor grids) for at least 1 sec. The conditioned response to the CS was said to be freezing during the tone presentation minus freezing prior to the tone. For purpose of ranking the animals, CS freezing during the second training trial was used.

### Odor Discrimination (OD)

Rodents are adept at using odor to guide their search for food. In this task, mice navigated through a field using unique odors to guide them. The animals learned to choose a food cup that was signaled by a target odor among three odor choices. For this purpose, plastic food cups were used that held a cotton swab loaded with 25 µl of odor (anise, banana or coconut flavored extract). This swab was located at the bottom of the cup and was covered with a wire mesh. On each trial, the food cups were randomly arranged in three corners of the square test field, but accessible food was always marked by the target odor (in this case coconut).

The odor discrimination chamber consisted of a black Plexiglas 60 cm square field with 30 cm high walls located in a dimly lit room with good ventilation. One of three plastic food cups was placed in three corners. Only the target cup (marked by the coconut odor) had the food (30 mg portion of chocolate flavored puffed rice) accessible on top of the wire mesh. The other two cups had food located under the wire mesh, allowing the mice to smell the food but not access it.

Each animal had one day of acclimation and one day of testing. At the end of the light cycle on the day prior to this acclimation, food was removed from each animal's home cage. The next day each mouse was placed in the box for 20 min. with no food cups present. At the end of the day, each animal received three pieces of the reinforcer in their home cage. On the training day each animal received six trials in which they were placed in the corner of the training chamber which did not contain a food cup. On the first trial, an additional reinforcer was placed on the edge of the target cup (coconut). At the end of each trial the food cups and the starting location were rearranged but coconut always remained as the target odor. For each trial, the number of errors prior to retrieving food were recorded (where an error was constituted by making contact with or sniffing within 2 cm of an incorrect food cup). For purpose of this analysis, the average errors across trials two and three served to index learning.

### Brain Dissection

Two weeks following the completion of the learning battery the animals were sacrificed and their brains extracted. Specifically, the animals' were live decapitated according to standard animal ethical protocols and their brains quickly dissected to remove the prefrontal cortex. The tissue was immediately placed in a solution of RNAlater (Ambion) to preserve RNA integrity.

### RNA Isolation

Total RNA isolation followed the protocol recommended in the RiboPure RNA Isolation Kit (Ambion). Tissue samples were first homogenized in a TRI reagent solution and combined with 1-bromo-3-chloroprapane. The resulting mixture was centrifuged and the aqueous solution removed. The total RNA containing solution then underwent purification using glass fiber cartridges.

Each animal was assigned a factor scores derived from the principal component analysis (see Results, below), and these factor scores were used to determine each animal's aggregate performance across the battery of learning tests (i.e., to characterize the general learning ability of each animal). The resulting RNA from the four best learners were pooled and the same was done for the four worst learners. The total RNA was maintained at −70°C for storage (of several weeks prior to analyses). By using pooled tissue samples from the best and worst learners, spurious differences in gene expression (i.e., those not directly related to variations in general learning abilities) were likely to have been minimized.

### cDNA Synthesis and Microarray Hybridization

cDNA synthesis and microarray hybridization were carried out at the Keck Microarray Facility at Yale University (New Haven, CT). The gene expression analysis utilized the Illumina Sentrix MouseRef-8 BeadChip containing target probes for ∼25,000 annotated mouse genes.

As per the Keck Microarray Facilities procedures, the preparation of labeled cRNA for hybridization onto Illumina BeadChips followed the recommended Illumina protocol using a TotalPrep RNA Amplification kit (Applied Biosystems). Double stranded cDNA and biotin-labeled cRNA were synthesized and purified from 500 ng of total RNA. Purification of the cRNA followed, and integrity of the cRNA was assessed by running aliquots on the Bioanalyzer prior to hybridization.

Hybridization buffer from the BeadChip kit (Illumina) was mixed with 1500 ng of biotin-labeled cRNA, heated to 65°C for 5 minutes, and then loaded onto the BeadChip. The BeadChips were sealed in a hybridization chamber and placed in an oven at 58°C with a rocker for 16–20 hours. After the hybridization, the BeadChips were washed and stained as outlined in the Illumina protocol. The BeadChips were then scanned on the Illumina Iscan. Scanned files were loaded into BeadStudio software for analysis and arrays were background normalized.

### Gene Expression Quantification by QPCR

QPCR was carried out at the Burnham Institute (La Jolla, CA). Applied Biosystems Taqman probes were chosen for each of the 10 genes plus one house-keeping gene, GAPDH (Table 1). The probes chosen crossed at least one exon-intron junction so as not to be specific to any alternative splice forms. The cDNA was synthesized from 11 µl of total RNA (65–70 ng/µl) using Roche Trancriptor First-Strand cDNA Synthesis Kit. For QPCR 20 µl of cDNA was diluted to 60 µl (2 µl for each reaction). Taqman QPCR was performed using Taqman universal master mix (ABI Part # 4304437). All reactions were done in duplicate and relative concentrations values were calculated using a standard curve for known quantities of GAPDH.

**Table 1:** Applied Biosciences Taqman probe sequences used for QPCR.

Gene Symbol	Assay ID	NCBI Gene Reference	Probe Sequence
Drd1a	Mm01353211_m1	NM_010076.3	TGGTCTCCCAGATCGGGCATTTGGA
Slc25a18	Mm01183193_m1	NM_001081048.2	TGCTGGCCGCTTAGCTGTCTGTCAT
Ddx6	Mm00492142_m1	NM_181324.3	CAATCTTGTTTGCACTGATCTGTTT
Rgs9	Mm00599991_m1	NM_011268.2	CACCCAGCCAGGTCAGCACTTGGCT
Kcnh1	Mm00495110_m1	NM_010600.2	GAGAGAGAGTCAGGGCATCAGCAGC
Nudt6	Mm00463700_m1	NM_153561.2	AGATATTGACACAGCAGTCCGAGAG
Psmc3ip	Mm00464703_m1	NM_008949.2	TGGAGGCCGAGCTGAAGGAATTAAC
Ppp1r1b	Mm00454892_m1	NM_144828.1	CAGCAGGGGCACTGTGGGGCAGAAG
Scn1a	Mm00450580_m1	NM_018733.2	ACTGAAGGCTGTGTCCAGAGATTCA
Atp8a1	Mm00437713_m1	NM_001038999.1	GCAGAACCTGCTTCACGGCTATGCT

In addition to the 10 genes for which we quantified expression we also quantified one control/housekeeping gene, glyceraldehyde 3-phosphate dehydrogenase (GAPDH). This was done so as to both verify the efficacy of the QPCR as well as to control for differences in starting RNA concentrations by normalizing the expression values against this gene. However, it was found that GAPDH values were not equal between the fast learners and the slow learners. While there was not a significant relationship between GAPDH gene expression and general learning abilities, there was a negative correlation such that faster learners tended to express more GAPDH mRNA transcripts. Therefore using this gene to normalize the results would necessarily skew the results away from finding any relationship with general learning abilities. Due to this complication, we used the expression values for Psmc3ip to normalize the data. Psmc3ip was chosen because there was no relationship between the raw/unnormalized expression values for this gene and general learning abilities nor was there a relationship when this gene was normalized against GAPDH. Using Pscmc3ip to control for differences in starting RNA concentration, therefore, would most accurately represent the data.

## Results

**Figure 1:**
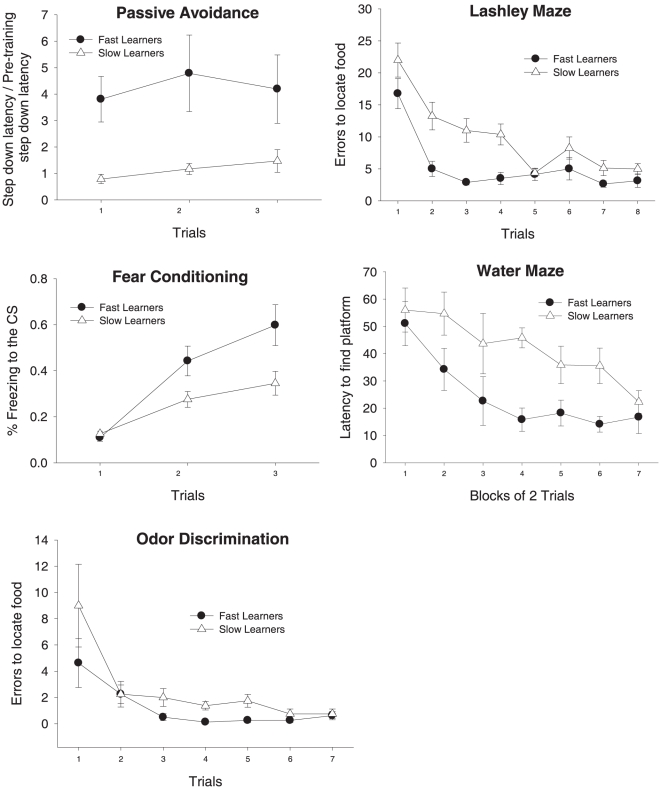
Sixty animals were assessed in a battery of learning tasks. Following testing on five learning tasks, the aggregate performance (factor scores) of each individual animal across all tasks was used as an index of their general learning abilities. The performance of the top and bottom eight animals from this distribution of general cognitive abilities are illustrated on each of the tasks. It was these eight fast and eight slow learners that contributed to the initial gene expression analysis. Based on these illustrations, it can be concluded that aggregate performance (general learning ability) is a good predictor of animals' performance on individual learning tasks. Brackets indicate standard error of the mean.

The original sample of 60 mice was divided into two independent biological replications of 30. A principle component factor analysis of learning performance on the battery of five learning tasks extracted a single stable factor in each of these two replications. This general learning factor explained between 41–42% of the performance variations in each of the learning tasks (Table 2). Although 30 subjects are generally considered small for factor analysis, the structure of resulting factor was very similar to the results of a previous analysis that included 241 animals [11]. Also, it is very similar to the results of intelligence batteries using human subjects where a general factor has typically been reported to explain from 38–50% of the underlying variance [18]. From this analysis, a general learning factor score was calculated for each of the animals (a factor score is analogous to an intelligence quotient in that it is a measure of where an animal falls on the observed distribution of general learning abilities). In this way we were able to identify both those animals with the best and the worst general learning abilities in each replication (Fig 1).

**Table 2:** Principal component factor analyses of the performance in the learning battery as well as gene expression values for the PFC genes identified through the microarray analysis as being differentially expressed.

Learning Tasks	Replication 1	Replication 2	Combined
Lashley Maze	0.79	0.73	0.76
Water Maze	0.62	0.67	0.64
Fear Conditioning	0.55	0.40	0.47
Passive Avoidance	0.57	0.90	0.77
Odor Discrimination	0.63	0.40	0.51
**Eigenvalue**	**2.03**	**2.12**	**2.05**
**% Variance Explained**	**41%**	**42%**	**41%**

Principal component factor analyses of the performance in the learning battery. Columns (replication 1, replication 2, and combined) show how each task loads on the general learning factor in each replication and in a combined analysis. The structure of the resulting general learning factor in each replication was stable and explained between 41–42% of the variance in performance in the learning battery.

Due to high individual variance in gene expression, RNA samples from the fastest learners were pooled, and similarly, the same was done for the poor learners. In doing so those genes that were differently expressed were less likely to reflect genes unrelated to animals general cognitive performance (i.e., false positives). Since the samples were pooled, no estimate of variance was possible, and therefore, a cut-off was chosen for the ratio of fast/slow learners' expression levels for each gene. This fold difference ( = 1.34, the limit of detection for the Illumina chip [based on Illumina technical report]) was used to classify genes as being differentially expressed in the two sample of tissue. Of the 24,000 genes whose expression was assessed in each replication, less than 0.03% were differentially expressed in the PFC (Table 3) across both replications. (Approximately 100 genes were differentially expressed in each replication. Of that 100, nine were present in *both* replications, and it is those nine that were designated as genes of interest.) Three of these genes, the dopamine D1 receptor (Drd1a), Rgs9, and Darpp-32, share a common functional pathway (dopamine signaling). To formalize whether the list of differently expressed genes belonged to a particular gene ontology (GO), we used the GOrilla tool to compare the differently expressed genes against all of the genes assessed by the gene chip. This analysis assigned the function ‘dopamine D1 receptor activity’ a significant p-value (p = 5.13E-4) [19]. (It is worth noting that of the all of the genes that were differentially expressed in the two replications, approximately 65% were up-regulated in the fastest learners. In contrast, when those genes that were differentially expressed only in *both* replications were isolated, *all* nine genes-of-interest were up-regulated in the fastest learners. At present, it is unclear if this uniform up-regulation represents a meaningful relationship between gene expression and intelligence or if it is simply a statistical anomaly.)

**Table 3:** Based on a fold change of at least 1.35 in both independent replications, 10 genes were identified as being differentially expressed in the PFC of mice that had exhibited fast relative to slow general learning performance.

Prefrontal Cortex			
Gene	Description	Direction of Regulation	Function
Atp8a1	Atpase	UP	ATP binding
Ddx6	DEAD (Asp-Glu-Ala-Asp) box polypeptide 6	UP	required for microRNA-induced gene silencing
Kcnh1	potassium voltage-gated channel, subfamily H (eag-related), member 1	UP	Delayed-rectifier potassium channel
Nudt6	nudix (nucleoside diphosphate linked moiety X)-type motif 6	UP	Trophic factor
Slc25a18	solute carrier family 25 member 18	UP	transport of glutamate across the inner mitochondrial membrane
Scn1a	sodium channel, voltage-gated, type I, alpha	UP	Pore forming unit voltage-gated sodium channel
Darpp-32	dopamine, cAMP-regulated phosphoprotein of 32,000 kDa	UP	phosphoprotein phosphatase inhibitor activity
Rgs9	regulator of G-protein signaling 9	UP	negative regulation of signal transduction
Drd1a	dopamine receptor D1A	UP	dopamine D1 receptor activity

**Figure 2:**
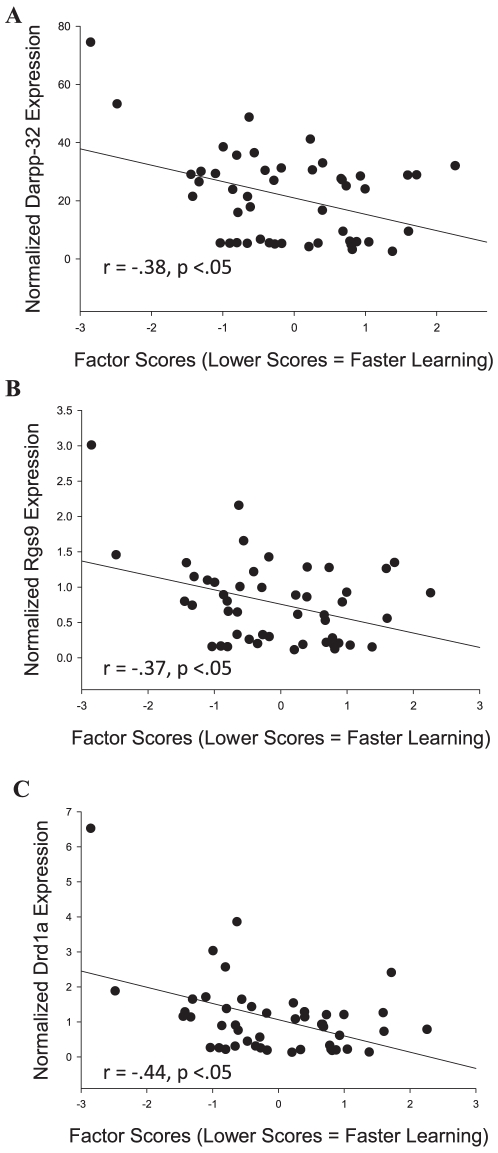
Correlations between normalized gene expression in the PFCs of 48 mice (y-axis) and their general learning ability factor scores, which is analogous to general intelligence in humans (lower scores  =  faster learning). Three dopamine-related genes showed significant negative correlations: **A**) Darpp-32, **B**) Drd1a, **C**) Rgs9.

**Figure 3:**
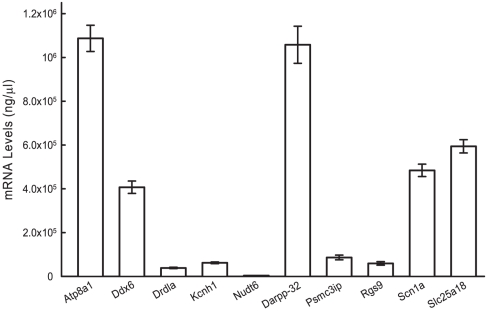
Overall gene expression in the prefrontal cortex of each gene whose expression was assessed with QPCR.

To further explore and validate these results, the expression values of the nine genes that were identified as being differentially expressed in the PFC were assessed in the top 24 and bottom 24 animals from the original 60 mice using quantitative real-time PCR. The expression values for these genes were then correlated with the animals' general learning factor scores. Of the nine genes, only four (Nudt6: r (46) = −0.29, p<.05; Darpp-32: r (46) = −0.38; Drd1a: r (46) = −0.37, p<.05; Rgs9: r (46) = −0.44, p<.05) were significantly correlated with general learning abilities. Importantly, the three genes related to dopamine signaling identified as elevated by the previous gene expression analysis were significantly correlated with general learning abilities (Fig. 2). It is worth noting that while Rgs9 is generally considered to be expressed primarily in the basal ganglia, it is expressed at low to moderate levels in the mouse prefrontal cortex [20]. We found similarly moderate Rgs9 levels here (6% of the Darpp-32 levels, see Fig 3). To account for possible non-parameteric relationships (as might be introduced by the high values of several data points) we also performed a Spearman's rank correlation (which mitigates the influence of extreme values by assessing the correlation of nominal ranks rather than actual raw values). Of the four genes found to be statistically correlated using Pearson's coefficient, Drd1a and Nudt6 were found to be statistically correlated with general learning abilities using Spearman's rank correlation (Drd1a: r(46) = −0.36, p<.01; Nudt6: r(46) = −0.35, p<.02). The remaining two genes (Rgs9 and Darpp-32) showed a trend towards significance (Rgs9: r(46) = −0.25, p<.09; Darpp-32: r(46) = −0.25, p<.09).

When the expression values of the PFC genes were included in a rotated factor analysis (a rotated factor analysis extracts the most number of uncorrelated factors) to determine their shared variance, two factors were extracted. A primary factor (dopamine-specific factor) accounted mostly for the variance shared by the three dopamine related genes while an uncorrelated second factor accounted for the variance shared by all the genes *minus* the dopamine specific variance (Table 4). In other words, the dopamine factor quantified the unique variance shared by the dopamine genes minus any shared variance they had with the rest of the genes. When the factor scores extracted from these two factors were included in a factor analysis with the learning battery performance data, only the dopamine specific variance loaded with the learning tasks (Table 5). Furthermore, the dopamine factor correlated significantly with the general learning factor (r (46) = −0.44, p<.05). This suggested that the variance common to the three dopamine specific genes *uniquely* predicted performance on the learning tasks.

**Table 4:** A maximal-likelihood rotated factor analysis including all of the prefrontal genes revealed a primary factor which accounts for the common variance shared by the dopamine-associated genes and secondary factor which explains the remaining variance.

Differentially Expressed PFC Genes	Dopamine Factor	Remaining Variance
Atp8a1	0.34	0.92
Ddx6	0.39	0.85
Drd1a	**0.78**	0.54
Kcnh1	0.38	0.88
Nudt6	0.24	0.75
Darpp32	**0.78**	0.56
Rgs9	**0.98**	0.15
Scn1a	0.24	0.89
Slc25a18	0.31	0.90
**Eigenvalue**	**2.81**	**5.18**
**% Variance Explained**	**31%**	**57%**

**Table 5:** A factor analysis including the learning tasks and the factor scores extracted from Table 3 revealed that the dopamine-associated genes share a unique relationship with the learning tasks.

Learning Tasksand Gene Clusters	General Learning Factor
Lashley Maze	0.49
Water Maze	0.35
Fear Conditioning	0.30
Passive Avoidance	0.97
Odor Discrimination	0.32
Dopamine Factor	**−0.68**
Remaining Variance	**−0.01**
**Eigenvalue**	1.99
**% Variance Explained**	28%

## Discussion

Here we observed a relationship between general intelligence (assessed in genetically heterogeneous mice) and dopamine signaling in the prefrontal cortex. To our knowledge this is the first time that such a direct relationship between a specific molecular pathway and general intelligence has been reported (although related analyses, based on peripheral tissue samples, have been conducted [21]). Specifically, it was determined that an up-regulation in three dopamine-related genes (Darpp-32, Rgs9, and Drd1a) was significantly correlated with animals' aggregate performance across a battery of learning tests. While it is perhaps premature to speculate about the functional consequence of an up-regulation in these three genes in faster learners, it seems likely that they interact to suppress the activation of protein phosphatase 1 (PP1) and thereby enhance synaptic plasticity and the efficacy of D1 mediated signaling. Activation of D1 dopamine receptors results in a cascade of events which phosphorylates Darpp-32, which in turn inhibits PP1 [21]–[22]. PP1 inhibits learning and memory by negatively regulating downstream proteins and kinases important for synaptic plasticity and by reducing neuronal excitability [23]–[25]. An increase in the main functional unit of D1 receptors (Drd1a) as well as Darpp-32 could therefore act in concert to decrease the suppression of learning and memory. Conversely, D2 dopamine receptor activation reduces phosphorylation of Darpp-32 which in turn releases inhibition of PP1. However, Rgs9 dampens the downstream effects of D2 activation [26]. Therefore, an increase in Rgs9 activity may also act to enhance the suppression of PP1. Via this route, D1-mediated dopamine signaling efficacy in the prefrontal cortex could act to enhance working memory function and therefore increase general intelligence. In support of this possibility, it is known that that during working memory tasks, activity of dopaminegic midbrain neurons is enhanced and dopamine levels in the prefrontal cortex increase [27]–[28]. Similarly, studies have shown that sequence differences in Darpp-32 in humans are associated with differences in Darpp-32 expression, increases in neostriatal volume, enhancements in connectivity between the striatum and the prefrontal cortex and better performance on both working memory tasks and general intelligence batteries [29].

Two of the genes that were differentially expressed in the prefrontal cortex, Rgs9 and Darpp-32, are studied primarily for their role in the basal ganglia where they exhibit orders of magnitude higher expression levels. Despite this, these genes have also been shown to be expressed in the cortex. Darpp-32 shows widespread cortical expression [30]. While Rgs9 is often thought to be specific to the basal ganglia, in mice it has been demonstrated to be expressed at low to moderate levels in the cortex [20], [31]. Nevertheless, it is possible that the present results also suggest changes in basal ganglia-related pathways as being related the general learning abilities. This seems plausible as there are large bidirectional pathways linking the PFC and the basal ganglia. Relatedly, most models of selective attention suggest a reciprocal role for both the basal ganglia and the PFC [32]. In fact, fMRI studies have demonstrated that activity levels in the PFC and the basal ganglia are increased prior to filtering of irrelevant information in selective attention tasks. Lastly, the degree to which both these regions were activated predicted individual differences in working memory capacity [33].

It is worth noting here that a large body of research has demonstrated that D1 mediated dopamine signaling plays a crucial role in facilitating learning early in acquisition but that its role is diminished as the animals' performance reaches asymptotic levels [30]. This finding is consistent with the present results as we only detect a general learning factor in our mice when we examine learning at the early stages of acquisition, a point at which differences in D1 signaling seem to be involved [11]; [34].

Dopamine signaling in the PFC increases the neural excitability of pyramidal neurons, increasing their gain in response to excitation. Models of PFC neurons' persistent firing during a working memory task have demonstrated that this dopamine-mediated increase in gain acts to stabilize persistent activity and protect it from interference [35]. This finding fits with the hypothesized role of the prefrontal cortex in working memory and in general intelligence. That is, the PFC acts to maintain attention towards goal relevant information and to ignore salient distracters. In light of this model our findings integrate nicely into the existing general intelligence literature using human subjects. Similarly, these results fit with our previous behavioral work that demonstrated a significant relationship between working memory capacity/selective attention and general learning abilities [15]–[16].

Independent of the three dopamine-related genes, only one other gene, Nudt6 (also known as basic fibroblast growth factor; bFGF), showed a significant correlation with general learning abilities. This gene is expressed by astrocytes where it acts as a potent trophic factor for neurons [36]. In culture bFGF has been shown to promote the survival of prefrontal cortical neurons [37]. Outside the brain, bFGF has been shown to promote angiogenesis [38]. In addition, bFGF is up-regulated in mice that underwent voluntary wheel running for 4 days as compared to sedentary controls, implicating this gene in the positive cognitive effects of exercise [39]. The potential implications of the upregulation of this gene for general learning abilities are two-fold. It is possible given the relatively low levels of bFGF found in our samples that the differences were indicative of differential levels of prefrontal vascularization in fast and slow learners. Poor blood flow would have obvious detrimental effects on cognitive performance. This is demonstrated by the correlation between age-related cognitive decline and cerebral blood-flow [40]–[41]. The second possibility is that the direct trophic effect bFGF exerts on neurons enhances neuronal survival in fast learners. This may be directly related to general learning abilities (e.g., enhanced survival increases the efficacy of synaptic plasticity). Conversely, it may be a secondary effect of potentially increased neuronal activity that may accompany fast learning abilities. For instance, enhanced activity of PP1 through Darpp-32 phosphorylation could exert stress on neurons, as when activated, PP1 works to conserve energy through a recycling of protein factors, and the reversal of the cell to an energy-conserving state [23]. In turn trophic factors, such as bFGF, may be needed to maintain cell survival in face of this increased stress.

It is also important to note that the nature of the present study was correlative and therefore not able to discern the direction of any potential casual relationships. It is conceivable that, for instance, poor general learning abilities resulted directly in reduced dopamine expression in the PFC or that poor learners were more sensitive to environmental factors affecting dopamine related pathways. To directly test these possibilities are beyond the scope of the present study. However, the veracity of these alternate hypothesizes appear unlikely given that there is a large body of literature suggesting a close causative relationship between working memory and PFC dopamine and that we recently demonstrated a direct causative relationship between working memory and general learning abilities in mice [42]. Nevertheless, appropriate caution must be taken in drawing any broad conclusions from the current results.

In total, these results implicate a small number of genes, particularly a cluster related to dopamine D1 signalling in the PFC, as being related to and potentially as being mediators of general cognitive abilities (c.f., general intelligence). It is critical that these results *not* be interpreted to suggest that *only* these genes contribute to this regulation. As a first approximation, here, methods were used that maximized sensitivity to dominant genes while minimizing the likelihood of false positive gene identification. It is likely that additional research will identify more and more complex interactions between a compendium of genes involved in the regulation of this complex cognitive trait.
